# A Review of Novel Oximetry Parameters for the Prediction of Cardiovascular Disease in Obstructive Sleep Apnoea

**DOI:** 10.3390/diagnostics13213323

**Published:** 2023-10-26

**Authors:** Siying He, Peter A. Cistulli, Philip de Chazal

**Affiliations:** 1 Charles Perkins Centre, Faculty of Engineering, Sydney University, Camperdown, NSW 2050, Australia; sihe9643@uni.sydney.edu.au; 2 Charles Perkins Centre, Faculty of Medicine and Health, Sydney University, Camperdown, NSW 2050, Australia; peter.cistulli@sydney.edu.au; 3 Department of Respiratory and Sleep Medicine, Royal North Shore Hospital, St Leonards, NSW 2065, Australia

**Keywords:** sleep apnoea, cardiovascular disease, pulse oximetry, hypoxia

## Abstract

Obstructive sleep apnoea (OSA) is a sleep disorder with repetitive collapse of the upper airway during sleep, which leads to intermittent hypoxic events overnight, adverse neurocognitive, metabolic complications, and ultimately an increased risk of cardiovascular disease (CVD). The standard diagnostic parameter for OSA, apnoea–hypopnoea index (AHI), is inadequate to predict CVD morbidity and mortality, because it focuses only on the frequency of apnoea and hypopnoea events, and fails to reveal other physiological information for the prediction of CVD events. Novel parameters have been introduced to compensate for the deficiencies of AHI. However, the calculation methods and criteria for these parameters are unclear, hindering their use in cross-study analysis and studies. This review aims to discuss novel parameters for predicting CVD events from oximetry signals and to summarise the corresponding computational methods.

## 1. Introduction

Obstructive sleep apnoea (OSA) is a sleep disorder caused by repeated collapse of the upper airway during sleep. It is more commonly observed in patients over 40 years old, with a larger body mass index (BMI), or who have a narrow airway and a unique facial structure [1]. Patients with OSA have a higher chance of developing depression, cardiovascular disease (CVD), and diabetes, and of having car accidents [2]. The OSA-induced repetitive upper airway obstruction leads to intermittent hypoxic events overnight and sleep fragmentation, resulting in adverse neurocognitive, daytime sleepiness, and metabolic complications [3]. The nocturnal hypoxemic burden caused by cumulative hypoxic events can increase vascular inflammation, blood pressure, and sympathetic nervous system action, and ultimately may increase the risk of CVD, which is the leading cause of death worldwide [4,5]. Studies have shown that OSA is associated with CVD morbidity and mortality, with 43–73% and 47–76% of CVD cases having OSA [6,7,8].

Overnight polysomnography (PSG) is commonly used for OSA diagnosis with the apnoea–hypopnoea index (AHI) being the standard measure for determining the presence and severity of OSA. PSG signals record blood oxygen level (measured with finger-based pulse oximetry), respiratory pressure/flow and effort, brain activity, skeletal muscle activity, heart rate, and eye movements. The AHI measures the number of apnoea and hypopnoea events per hour of sleep [9,10,11,12,13]. However, studies show that AHI is not a good predictor of CVD mortality as AHI fails to capture factors that have crucial impacts on the cardiovascular system, namely, blood oxygen levels, high sympathetic activity, respiratory event duration, sleep fragmentation, and arousal events [14,15,16].

As the understanding of the links between CVD and sleep apnoea has grown, new PSG-based parameters have been proposed that may reveal more information about the impact of sleep apnoea on hypoxemia that may be predictive of future CVD events [17].

Studies have shown that some oximetry parameters may provide good performance in the prediction of future CVD events. These parameters include T90, oxygen desaturation index (ODI), and area-based desaturation metrics [4,14,18,19,20]. The T90 parameter measures the time below 90% oxygen saturation; ODI indicates the number of oxygen desaturation events per hour of sleep; and area-based desaturation metrics calculate the area of the desaturation [14,19,20,21]. However, the calculation of each parameter varies between studies, which limits the cross-study comparison of results [4,19,20,21,22]. This review aims to present novel oxygen saturation parameters for predicting CVD morbidity and mortality, and to summarise the calculation methods used.

## 2. Literature Search Methodology and Outcomes

A literature search using the databases of Medline via OvidSP and Scopus was performed up to 1 July 2023, and all prior years of publication were considered. The search strategy involved the following sleep apnoea and cardiovascular disease related keywords: oximetry, sleep apnoea, and cardiovascular. We then filtered the papers according to the following criteria: studies were in adult humans only; the study focused on the association between OSA and CVD events; the article either described the computational methods in detail or cited articles with such content; and the study population was greater than 200 participants.

Our literature search yielded 115 publications which we grouped into 4 categories based on oximetry parameters. These parameters were time below 90% saturation (T90), oxygen desaturation index (ODI), desaturation area-based parameters, and other parameters. We provide an in-depth discussion of the methods of the first 3 categories and a summary discussion of the 4th category. A total of 30 articles used T90, 31 articles explored ODI, 43 articles used desaturation area-based parameters, and 11 articles considered other parameters.

A further selection was then performed on the articles for a detailed analysis of CVD predictive performance. We selected articles that used multiple parameters for comparison on the same study population, and/or considered novel CVD outcomes. This selection yielded 28 publications with 11 papers focused on T90, 6 papers using ODI, and 11 papers delving into desaturation area-based parameters.

## 3. Time below 90% Saturation

Time below 90% saturation (T90) is recognised as an independent predictor of all-cause CVD mortality and widely used in many studies [4,23,24,25,26,27]. The calculation method of T90 varies among studies and can be divided into time-based and percentage-based parameters, as shown in Table 1 [4,14,18,22,27,28]. The time-based TST90 measures the time per night with oxygen saturation below 90%, and thus measures the cumulative hypoxemia insult. The percentage-based T90% measures the percentage of the time per night below 90%, and thus measures the hypoxemia insult rate. Both methods perform well in predicting CVD events. Xu et al. concluded that time-based TST90 is a robust predictor of major adverse cardiovascular events (MACEs) and performs much better than AHI [27]. Baumert et al. categorised time-based T90 according to the proximity of the dips below 90% to desaturation events. T90desaturation is the time spent below 90% oxygen saturation associated with acute desaturation patterns of at least 4%. T90non-specific is the time spent below 90% oxygen saturation associated with non-specific drifts. Both were shown to be good indicators of the association between OSA and CVD mortality. The researchers suggested that the performance of CVD mortality prediction can be improved by using T90desaturation and T90non-specific as multivariate inputs [4]. Percentage-based T90% is a reliable predictor of CVD events too. Wang et al. found that T90% outperformed TST90 in the prediction of incident CVD in patients with non-sleepy sleep-disordered breathing (SDB) [18]. However, other studies have not supported this conclusion. Sutherland et al. found no significant association between T90% and incident CVD [29] in OSA patients. It is currently unclear whether TST90 or T90% is a better CVD predictor. Further research and comparison of T90 and T90%’s performance in different databases may be required.

**Table 1:** Different calculation methods of T90. Examples of each method with the corresponding database, the aim of analysis, and results are provided.

Type of T90	Name of T90	Calculation Method	Population/Aims
Time-based parameter	TST90 [27]	The total sleep time below 90% oxygen saturation. The unit of this parameter is time.	Study population: 1860 Chinese participants from a clinic-based retrospective cohort study in Hong Kong, China. Participants were excluded from study if they had a sleep disorder other than OSA; received treatments other than CPAP; or had conditions with a known effect on OSA. Aims: Association between T90 and MACEs.
	T90desaturation [4]	The time spent below 90% oxygen saturation associated with acute desaturation patterns of at least 4% as shown in Figure 1A. The unit of this parameter is time.	Study population: 3135 community-dwelling male participants aged 65 years old and above from the MrOS sleep study. Aims: Association between T90desaturation and CVD mortality.
	T90non-specific [4]	The time spent below 90% oxygen saturation associated with non-specific drifts as shown in Figure 1B. The unit of this parameter is time.	Study population: 3135 community-dwelling male participants aged 65 years old and above from the MrOS sleep study. Aims: Association between T90desaturation and CVD mortality.
Percentage-based parameter	T90% [18]	The percentage of sleep time with oxygen saturation level below 90%. The unit of this parameter is %.	Study population: 3626 randomly selected Chinese community-dwelling participants. A total of 30.7% of the participants suffer from SDB, of which 96.5% is non-sleepy SDB. Aims: Association between T90% and CVD incident in non-sleepy SDB patients.

**Figure 1:**
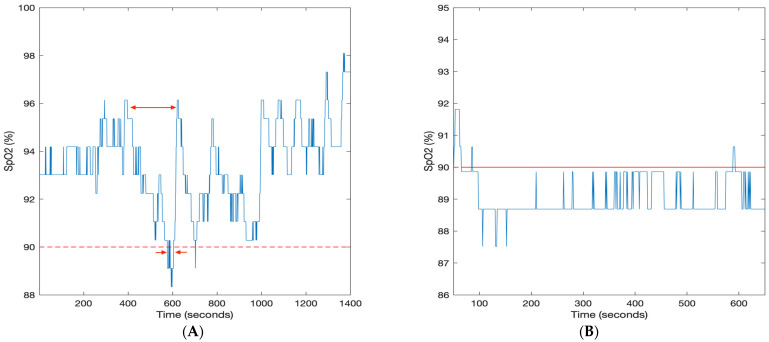
Pulse oximetry trace (SpO_2_) from the MrOS database. (**A**) T90desaturation is the time spent below 90% oxygen saturation associated with acute desaturation patterns of at least 4%. The long arrow indicates a desaturation event and the short arrows indicate the time below 90% for the event. (**B**) T90non-specific is the time spent below 90% oxygen saturation associated with non-specific drifts [4].

## 4. Oxygen Desaturation Index

Oxygen desaturation index (ODI) is commonly used to indicate intermittent hypoxemia, and is defined as the number of oxygen desaturation events per hour of sleep [14]. Although ODI and AHI both measure event rates, ODI performs better in predicting adverse CVD outcomes [30,31]. ODI measures the number of transient desaturation events from a baseline value and divided by the hours of sleep. The American Academy of Sleep Medicine (AASM) does not specify the criteria for scoring desaturation events [31,32,33,34,35], and hence a range of methods have been used to calculate ODI. Some studies define ODI as the rate of oxygen desaturation events occurring when SpO_2_ drops lower than the desaturation threshold from the average saturation in the previous 120 s and persists for at least 10 s [36,37]. When events are separated by less than 120 s (as it happens on average for a severe sleep apnoea case), the baseline will be influenced by previous events. Other studies chose the baseline as either the average SpO_2_ value of the whole recording or the mean SpO_2_ value in the first 3 min [38,39,40,41]. The desaturation thresholds of 3% (ODI3) or 4% (ODI4) are commonly chosen in the analysis of OSA and CVD [33,42,43]. Sutherland et al. provided a comparison of ODI2, ODI3, ODI4, and ODI5 for predicting prevalent CVD in OSA patients free of CVD at baseline, and found that 4% and 5% provide the best performance in predicting CVD events in women [29,44]. Karhu et al. concluded that ODI4 is more reliable than ODI3 in determining the impact of OSA, since respiratory events with desaturation ≥ 4% are usually considered as hypopnoea [21,33]. However, results from several studies showed that ODI3 as a CVD risk factor has a higher significant odds ratio (OR) than ODI4 [45,46]. Further research undertaken by Punjabi et al. explored whether ODIs within a specific range (2–2.9%, 3–3.9%, and 4–4.9%) are associated with CVD events. The results showed that only ODI (4–4.9%) is statistically significant in the analysis, and supported Tuomas et al.’s findings on ODI4 [44]. The hardware and software used to measure ODI metrics also impacts the ODI parameter. There was a clinically significant difference between the ODI measurements from the same database measured using the ResMed ApneaLink Plus device (ResMed, Sydney, Australia) and the Compumedics Grael Profusion PSG3 system (Compumedics Limited, Abbotsford, Victoria, Australia) [34]. Ng et al. suggested that this discrepancy may be caused by the noise cancellation process rather than the ODI scoring algorithm [34].

## 5. Desaturation Area-Based Parameters

Recent studies introduced novel parameters as promising indicators of future CVD events. These parameters calculate the area above the SpO_2_ curve associated with key sleep disorder breathing events per hour of sleep, and are distinguished by their different calculation methods. The units of these measures are %, and thus provide a weighted average value of the SpO_2_ trace. This group of parameters can be categorised according to their dependence on the respiratory event scoring. Hypoxic burden and respiratory event desaturation transient area are calculated based on manually scored respiratory events, while hypoxic load and desaturation severity are independent of respiratory events [14,47,48,49].

### 5.1. Hypoxic Burden

Azerbarzin et al. proposed hypoxic burden (HB) which is defined as the sum of the area between the SpO_2_ trace and the desaturation baseline associated with all apnoea and hypopnoea events divided by the total time of sleep, as shown in Equation (1) [14]:

(1) $$HB=\frac{\begin{array}{c} events\;\\ \sum area\;of\;an\;individual\;desaturation\;event \end{array}}{\sum time\;of\;sleep}.$$

**Figure 2:**
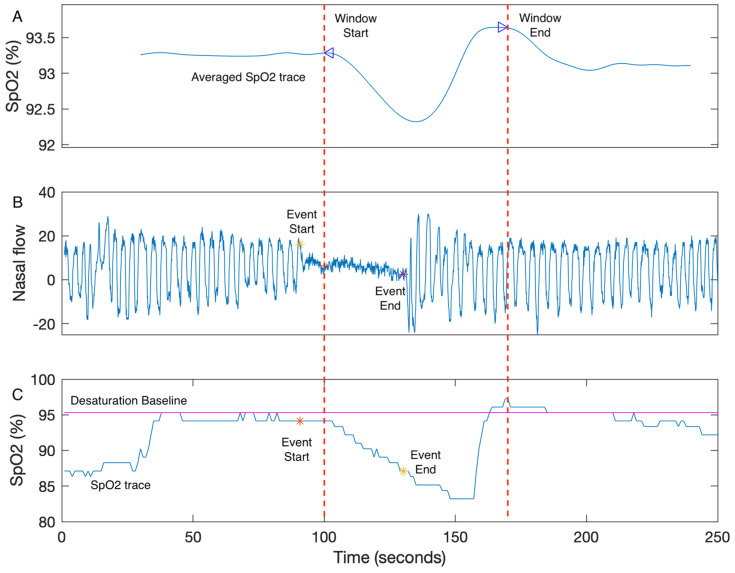
The example of HB calculation. (**A**) The search window is defined as the two peaks of the averaged SpO_2_ trace. (**B**) The nasal flow (blue) and the end points of a respiratory event (event start: yellow star; event end: purple star) are shown. (**C**) The SpO_2_ trace of the corresponding respiratory event is shown. The desaturation area for a single event is the area above the SpO_2_ trace (blue), below the desaturation baseline (magenta), and within the search window (between window start and window end). The desaturation baseline is the maximum SpO_2_ value within 100s prior to the event end (yellow star). HB is calculated as the sum of desaturation events divided by the total time of sleep [14].

The authors present the unit of HB as %minutes per hour of sleep which is equivalent to a measure with units of % scaled by a factor of 60. The calculation for HB can be decomposed into three main steps: (1) All SpO_2_ segments associated with manually scored respiratory events for one individual recording are averaged and processed to calculate the boundaries of a search window. The search window boundaries are determined by the two peaks of the averaged respiratory event as shown in Figure 2A. (2) The desaturation baseline for each respiratory event (Figure 2B) is calculated as the maximum SpO_2_ value within 100 s prior to the end of the event (Figure 2C). (3) The desaturation area for a single respiratory event is the area within the search window, desaturation baseline, and the SpO_2_ trace, as shown in Figure 2C. HB is then calculated using Equation (1) [14]. Other researchers have attempted to replicate HB with varying success. Trzepizur et al. [50] developed their own algorithms for HB but post hoc analysis by Mehra and Azerbarzin [14] suggested Trzepizur et al.’s method underestimated HB [51]. Based on the published material in [14], the algorithm was replicated by de Chazal et al., and the MATLAB code is publicly available in the online sharing platform GitHub (https://github.com/pdechazal/Hypoxic-Burden (accessed on 16 October 2023)). Two other commercial software packages calculate HB, Respironics (Murrysville, PA, USA) and Cidelec (Sainte-Gemmes-sur-Loire, France) [52]. However, the lack of a full disclosure of the algorithmic details of HB by the original authors has led to some confusion in the reproducibility of the HB calculation.

Researchers suggest that HB has a better performance than AHI in predicting CVD mortality and morbidity as it measures more information about the depth and duration of desaturations associated with apnoea and hypopnoea events [50,53,54,55]. Azarbarzin et al. conducted analysis on two population groups and demonstrated that HB has uniformly good performance for predicting CVD mortality in the two groups [14]. Blanchard et al. explored the correlation between OSA and stroke incidences using the database of the Pays de la Loire Sleep Cohort, and concluded that HB was a significant predictor of CVD events [53]. Trzepizur et al. compared the performance of ODI, T90, and HB in predicting MACEs, and concluded that T90 performs the best, while HB also proved to be a promising predictor [50]. However, HB outperformed T90 in predicting CVD mortality in patients from the Sleep Heart Health Study (SHHS) [47]. The varied conclusions regarding the performance of T90 and HB may be caused by the differences in database and target CVD events. As T90 and HB present different information derived from the SpO_2_ trace, future research could consider T90 and HB as multivariate predictors of CVD events.

### 5.2. Respiratory Event Desaturation Transient Area

**Figure 3:**
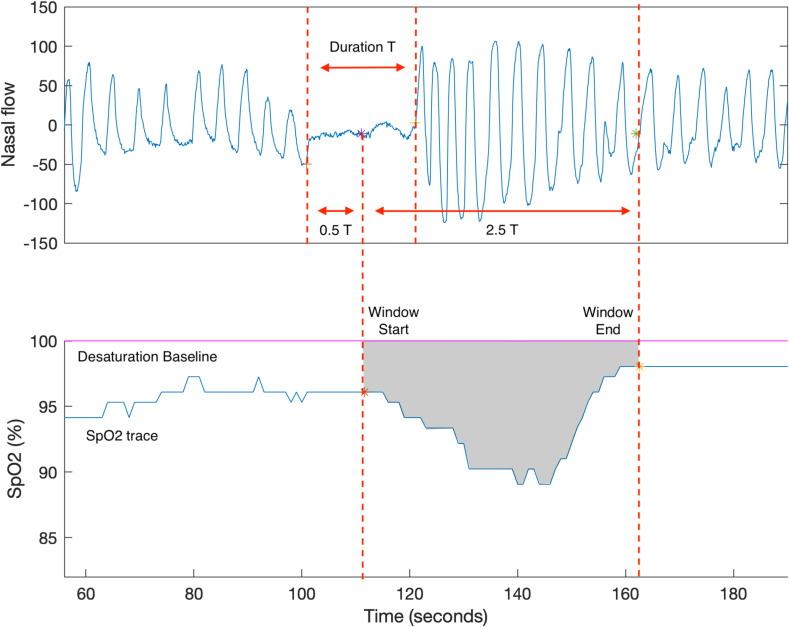
The example of REDTA calculation. (**A**) The nasal flow and a respiratory event are shown. The search window starts at the midway of the respiratory event and extends for 2.5 T, where T is the event duration. (**B**) The SpO_2_ trace of the corresponding respiratory event is shown. REDTA is calculated as the sum of the area (grey) within the search window, SpO_2_ trace, and the 100% desaturation baseline divided by 3600 [47].

Studies note that HB has limitations when used for some noisy recordings or recordings with few respiratory events. The accurate calculation of the desaturation area is challenging, as the desaturation baseline and the onset or offset of the average desaturation response are susceptible to noise. Moreover, the desaturation baseline is difficult to estimate when the interval between desaturation events is less than 100 s [47]. Our group proposed respiratory event desaturation transient area (REDTA) as a novel desaturation area-based parameter, which is less sensitive to noise than HB and has good predictivity of long-term CVD outcomes. REDTA is defined as the sum of the area between the SpO_2_ trace and the 100% desaturation baseline for all manually scored respiratory events divided by 3600, as shown in Equation (2):

(2) $$REDTA=\frac{\begin{array}{c} events\;\\ \sum area\;of\;an\;individual\;desaturation\;event \end{array}}{3600},$$

where the unit of each desaturation area is %seconds and the unit of REDTA is % hours [47]. REDTA is calculated using three main steps: (1) The search window is fixed and starts from the midway through the event and extends for 2.5 times the event duration. The search window is population-based (derived from the SHHS study) and is assumed to be appropriate for all respiratory events. (2) The desaturation area for a single respiratory event is the area between the 100% desaturation baseline and the SpO_2_ trace within the search window, as shown in Figure 3. (3) REDTA is the sum of the desaturation area divided by 3600. REDTA does not include the total time of sleep in the calculation. Its value increases with longer desaturation duration, more desaturation events, and greater depth of desaturation [47], and it thus is a measure of the hypoxemia insult per night. The software ABOSA (Version 1.1) implements REDTA [56]. The unit of REDTA is % hours.

REDTA was proposed to provide a simple, reproducible area-based SpO_2_ measure [47,57,58,59]. Pahari et al. and de Chazal et al. compared the ability of T90, ODI3, HB, and REDTA in predicting CVD mortality, and concluded that REDTA performed equivalently to HB and outperformed ODI3 and T90 in predicting CVD mortality [47,60]. Further work investigating REDTA and other CVD events is required.

### 5.3. Desaturation Severity

**Figure 4:**
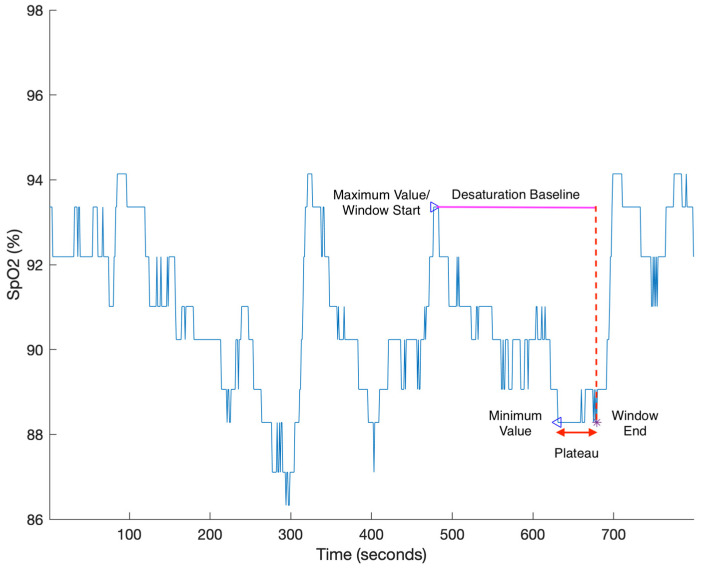
DesSev is calculated as the sum of the desaturation area divided by the total time of sleep. The desaturation area is calculated as the area between the desaturation baseline (magenta) and the SpO_2_ trace (blue) within the search window. The search window is to be defined as the time between the maximum and minimum values (blue triangles). However, due to the presence of a plateau (red arrow), the end of the search window is shifted forward to the end of the plateau (purple star). The desaturation baseline is the maximum value associated with the event [56].

Unlike HB and REDTA, desaturation severity (DesSev) does not use the respiratory events to calculate the area. The software package ABOSA implements DesSev and is freely available for other researchers to use [56]. DesSev is defined as the sum of the desaturation area associated with SpO_2_ events with a saturation drop greater than 3% divided by the total time of sleep [20,56]. The calculation of DesSev can be divided into three steps: (1) The potential start and end points of desaturation events are approximately identified. The start point is the peak of the SpO_2_ signal, and the end point is located at the minimum of the SpO_2_ signal, with at least 5 s between start and end point. (2) The start and end points are matched to form the candidate desaturation event list. The desaturation events are selected from the candidate desaturation event list based on four criteria: the event duration does not exceed 180 s; desaturation events do not overlap; if the flat plateau is longer than 30 s, the corresponding end point moves up to the end of the plateau; and the transient drop of desaturation event is greater than 3%. (3) As shown in Figure 4, the desaturation area is calculated as the area between the desaturation baseline and the SpO_2_ trace within the search window, while the desaturation baseline is the value of the start point, and the search window is defined by the start and end points. DesSev is calculated using Equation (3):

(3) $$DesSev=\frac{\sum_{n=1}^{number\;of\;desaturation\;events}{Desaturation\;area}_{n}}{\sum time\;of\;sleep},$$

and the unit of DesSev is % [56].

Studies have used DesSev to explore the association between OSA, CVD events, and cardiac response, and have concluded that DesSev is an informative indicator of OSA and cardiac response [61,62,63,64,65,66,67]. Kainulainen et al. concluded that there is a stronger association between average daytime sleepiness latency and DesSev than AHI or ODI. They suggested that excessive daytime sleepiness is more related to the depth and duration of desaturation events rather than to the number of desaturation events [65,66,67]. Associations between DesSev and the short-term time- and frequency-domain HRV parameters have been explored, and authors concluded that there is a significant association between DesSev and HRV in OSA patients [63]. DesSev may have some key limitations when applied to OSA patients. Because DesSev is independent of respiratory events, the desaturation area associated with respiratory events may be overestimated due to incomplete recovery from prior desaturation or non-OSA-induced hypoxemia [52]. To improve the accuracy of respiratory event-related desaturation severity estimation, Kulkas et al. introduced obstruction severity (*ObsSev*), later renamed sleep breathing impairment index (*SBII*) by Cao et al., which links *DesSev* to hypopnoea and apnoea events, as shown in Equation (4):

(4) $$ObsSev=SBII=\frac{\sum_{n=1}^{number\;of\;Hyps}{HypDur\times Desaturation\;area}_{n}+\sum_{n=1}^{number\;of\;Aps}{ApDur\times Desaturation\;area}_{n}}{\sum time\;of\;sleep},$$

where *Hyps* is the number of hypopnoea events, *Aps* is the number of apnoea events, *HypDur* is the duration of a single hypopnoea event, and *ApDur* is the duration of a single apnoea event. The unit of *ObsSev* (*SBII*) is %seconds [20,68]. The authors suggested that as *ObsSev* (*SBII*) captures more respiratory event information than other conventional SpO_2_ parameters, it may better predict OSA-related CVD outcomes [20,68]. Investigators have also found that *ObsSev* (*SBII*) is more age-related than AHI, and therefore can be used to estimate long-term CVD progression [69].

### 5.4. Hypoxia Load

**Figure 5:**
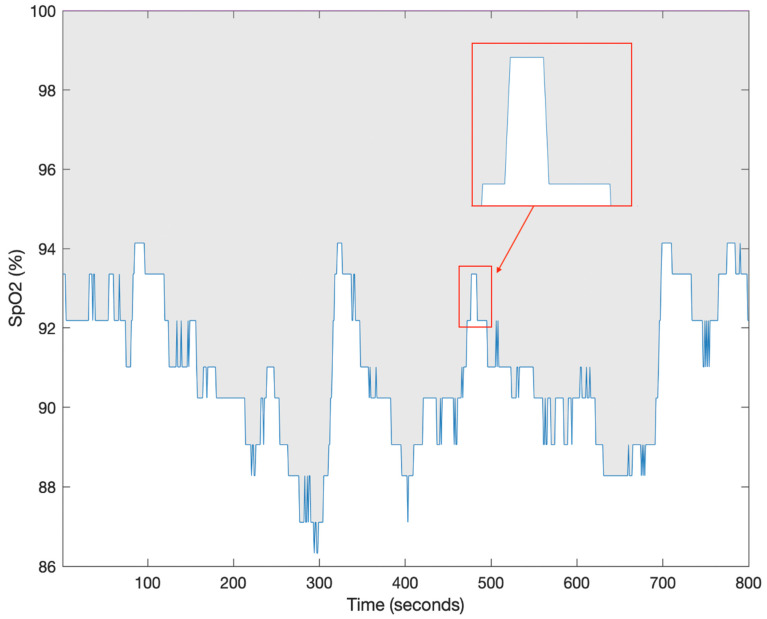
HL is calculated as the integrated area (grey) between the 100% baseline and the SpO_2_ trace divided by the total time of sleep [49].

Hypoxia load (HL) differs from other desaturation area-based parameters, as it is independent of any desaturation threshold or respiratory events. As shown in Figure 5, HL is defined as the desaturation area above the SpO_2_ trace divided by the total time of sleep. The calculation of HL can be divided into two steps: (1) The SpO_2_ saturation area is calculated by the numerical integration of the SpO_2_ trace using the trapezoidal rule, as shown in Equation (5):

(5) $$\;\int_{0}^{total\;time\;of\;sleep}{Area}_{SpO2}\approx \sum_{n=1}^{N}\frac{\left({SpO2}_{n}+{SpO2}_{n+1}\right)\times {(t}_{n+1}-{\;t}_{n})}{2},$$

where SpO2_n_ and SpO2_n+1_ are successive samples of the SpO_2_ trace, and the unit of the integrated area is %seconds [49]. (2) The average saturation is then calculated by dividing this area by the sleep time. HL is calculated using Equation (6). It is worth noting that the average saturation values in HL are also most exactly equal to the average SpO_2_ value during sleep reported by most PSG analysis software packages, the only difference being that the trapezoidal rule is used to calculate the area for HL whereas the average value use the rectangular rule to calculate the area.

(6) $$HL=100\%-\frac{saturation\;area\;during\;sleep}{\sum time\;of\;sleep}\approx 100\%-average\;SpO2\;value\;during\;sleep,$$

The unit of HL is % [49]. Because HL, unlike all previously discussed parameters, is not affected by any desaturation event criteria and thresholds, it is less likely to be miscalculated, which is ideal for cross-study analysis. However, the limitations of HL are also prominent: the information revealed by HL is not specific to transient changes in the SpO_2_ and may down-play the importance of OSA-related oxygen transients.

HL has not been widely used in the prediction of CVD events, but its association with other OSA-related symptoms has been explored. HL has been shown to be an independent predictor of fasting blood glucose and haemoglobin A1c (HbA1c) levels [70]. Linz et al. demonstrated that HL is also significantly correlated with CVD indicators in OSA patients after acute myocardial infarction, whereas AHI and other traditional metrics are not [49]. Similarly, Khoshkish et al. found a strong association between HL and blood pressure, while the conventional metrics of hypoxemia do not show an association. Although the correlation between nocturnal systolic blood pressure (BP) and HL became insignificant after adjusting for BMI, HL was strongly associated with the pulse pressure before and after the adjustment for BMI. It was suggested that HL is a suitable marker of BP patterns [71,72]. Considering that hypertension and diabetes are all associated with OSA, future studies using HL may reveal further associations between OSA and CVD [73,74,75,76].

## 6. Other Parameters

In studies exploring the associations between OSA and CVD outcomes, several other parameters derived from pulse oximetry have been investigated. Time domain parameters, such as mean SpO_2_, variance of SpO_2_, skewness of SpO_2_, and kurtosis of SpO_2_, were used measuring OSA characteristics [77,78,79,80,81,82,83]. Power spectral density as a frequency domain parameter, and sample entropy and central tendency measure as non-linear parameters were also considered in the OSA analysis [38,84,85,86]. These parameters are involved in the automated detection of OSA and infrequently investigated in CVD/OSA associations [29]. Sutherland et al. performed a comprehensive comparison of their performance in predicting incident CVD among OSA patients. The results showed no association between these parameters and incident CVD in men, with some parameters having marginal significance in the analysis of women [29]. These outcomes are based on a single study, and further studies on different databases are needed to confirm these results.

## 7. Summary of the Performance of Novel Oximetry-Derived Parameters in Predicting CVD Events

Table 2 summarises the performance of novel oximetry-derived parameters in predicting CVD events. Parameters that showed a statistically significant association with a CVD outcome ares listed in the second column. The third column lists the parameters that did not demonstrate a statistically significant association with a CVD outcome. 

## 8. Discussion

AHI remains the gold standard parameter for determining the OSA severity in clinical practice, but has shown poor performance in predicting future CVD events in OSA patients [97]. AHI focuses only on the frequency of apnoea and hypopnoea events, and assumes that all respiratory events have the same impact on OSA. It fails to show inter-individual differences in pathological effects of respiratory events and to present more information on the characteristics of the associated oxygen desaturation and cortical arousals [98]. Muraja-Murro et al. demonstrated that AHI after adjusting for duration of obstruction was better than AHI in predicting CVD mortality and morbidity. Azarbarzin et al. found that AHI was insufficient to predict heart failure in men, and that T90% and HB outperformed AHI in the prediction of all-cause mortality [14,54]. Not only did AHI prove to be inferior to T90% in predicting CVD mortality, but it also had limited effectiveness in predicting hypertension [88]. Butler et al. stated that other parameters derived from overnight PSG, such as respiratory event duration, could predict CVD events beyond AHI [15]. These suggest the need for parameters beyond AHI with other information when analysing the association between OSA and CVD [99].

As the understanding of the links between CVD and sleep apnoea has grown, parameters based on oximetry have emerged as an area to explore for developing PSG-based parameters predictive of future CVD events. Currently, there is no single oximetry parameter that is predictive of all event types. As shown in Table 2 HB, T90desaturation and REDTA are proven to be the most effective in predicting CVD mortality, while T90non-specific and ODI4 have insignificant hazard ratios in the analysis. TST90 works well in the prediction of MACEs, hypertension, diabetes, and endothelial dysfunction, some of which are pre-symptoms of CVD events. T90% outperforms other parameters in predicting right ventricular stroke, metabolic syndrome in women, and incident CVD in patients with non-sleepy SDB, while T90non-specific is recommended in the prediction of pulmonary hypertension. Although Karhu et al. proposed that ODI4 is more reliable than ODI3, more recent studies suggest that ODI3 is superior in predicting coronary plaque burden, the severity of OSA, and specific CVD risks [21,33]. HB has good performance in the prediction of stroke incidence, incident heart failure, and MACE, compared to other desaturation area-based parameters. DesSev and ObsSev/SBII differ from HB and REDTA in that they are independent of scored respiratory events. These parameters predict excessive daytime sleepiness, HRV, mean daytime sleep latency, acute stroke and transient ischemic attack, and CVD morbidity [58,59,67,68,96]. A few studies consider HL in their analysis. HL performs well at predicting HbA1c levels and blood pressure in patients with sleep disorders, which aids in diagnosing diabetes and hypertension.

Despite the significant results in the prediction of CVD events, most parameters fail to predict CVD outcomes in databases that includes OSA patients only. Sutherland et al. and Linz et al. found that TST90, T90%, ODI3, ODI4, and HB have insignificant predictions of incident CVD in OSA patients, and composite CVD outcomes in OSA patients with high CVD risks, respectively.

We see three main reasons for the varied results. First is the choice of database used to assess the parameters. Databases such as the SHHS are community sample which have a much greater representation oF controls than clinical samples or OSA-only samples, and it is perhaps unreasonable to expect a particular parameter to perform well across these distinct populations. We believe that the most useful populations to study these parameters in are clinical populations, because these are patient populations (by definition) seen in clinical practice. Second, baseline comorbidities and treatment programs need to be accurately recorded by studies. Third, without unambiguous and reproducible definitions of parameters, prediction performance may be impacted by individual implementations of parameters.

The definitions and calculation methods of each parameter provide different representations of the oximetry information content. For example, T90s focus on the duration of exposure to hypoxemia, whereas desaturation area-based parameters measure the transient hypoxemia associated with desaturation events. Until we have a complete understanding of the role of hypoxia in impacting CVD outcomes, a multivariate approach to CVD outcome prediction, which includes a range of oximetry and other PSG sensor parameters, will likely be a more successful approach than focusing on one particular parameter [100].

The main limitation of current studies is the inconsistent choice of computational algorithms, definitions, or databases, which hampers cross-study comparisons of results from the parameters. The lack of calculation standards for each parameter and the changing AASM criteria of standards affect the performance of parameters in predicting CVD events [101,102]. Moreover, the chosen databases may also influence the resultant outcomes. Some databases such as the SHHS were acquired decades ago and with advancements of sensor technology, results from these databases may have less relevance today. Some databases with a small sample size or referral bias compromise the generalizability of results derived from them [71,103,104,105,106,107]. Some studies used commercial software to calculate parameters, which lack uniformity and validation due to the unclear criteria for calculating parameters and different data processing techniques [34]. As we have focused on computational methods, we have not considered the level of evidence in the publications. In future studies, improvements could be made to address these issues: calculations or criteria for each parameter could be standardized; when determining the association between a parameter and CVD events, multiple analyses could be performed in different databases to reduce non-symptom-induced interference; and software-calculated parameters should be carefully considered for their accuracy and reliability.

## 9. Conclusions

This review discusses novel parameters derived from pulse oximetry for the prediction of CVD events, and summarises their corresponding computational methods and predictive results. These parameters fall into three main categories, namely, T90-based, ODI-based, and desaturation area-based parameters, each of which has its own strength in predicting particular CVD outcomes. We believe that standardized computational methods of parameters will help reduce some of the conflicting outcomes currently observed in the literature. As no single parameter is a stand-out predictor, we propose that advances in the prediction of CVD events in OSA patients may arise by considering multivariate SpO_2_ analysis.

## Figures and Tables

**Table 2:** The summary of AHI and oximetry-derived parameters and their performance in the prediction of CVD outcomes.

Parameter	Is a Predictor of	Is Not a Predictor of
AHI	All-cause mortality [87]	Incident heart failure [54] Composite CVD outcomes * [26] Hypertension [88]
TST90	MACE [27] Hypertension [89] Diabetes [89] Endothelial dysfunction [90]	Composite CVD outcomes ** [91]
T90%	Incident CVD in patients with non-sleepy SDB [18] Right ventricular stroke [92] Metabolic syndrome in women [93]	Incident CVD in patients with OSA only [29]
T90desaturation	CVD mortality [4]	
T90non-specific	Pulmonary hypertension [94]	CVD mortality [4]
ODI3	Coronary plaque burden [42] Severity of OSA [39] CVD risks *** [95]	Incident CVD in patients with OSA only [29]
ODI4		CVD mortality [4] Composite CVD outcomes * [91]
HB	CVD mortality [14] Stroke incidence [53] MACE **** [50] Incident heart failure [54]	Incident CVD in patients with OSA only [29]
REDTA	CVD mortality [47]	
DesSev	Excessive daytime sleepiness [59] HRV [58]	
ObsSev/SBII	Mean daytime sleep latency [67] Acute stroke and transient ischemic attack [96] CVD morbidity [68]	
HL	HbA1c level [70] Blood pressure [71]	

* Composite CVD outcomes include myocardial infarction, congestive heart failure, stroke, revascularization procedure, or death from any cause. ** Composite CVD outcomes (CVD death, stroke, myocardial infarction, heart failure, angina, transient ischemic event) in OSA patients with high CVD risks. *** CVD risks: the risks of CVD and CVD events including hypertensive disease, ischemic heart disease (IHD), cerebrovascular disease, diseases of arteries, arterioles, capillaries, and congestive heart failure (CHF). **** MACE: a composite outcome including all-cause mortality, acute myocardial infarction, stroke, and unplanned coronary revascularization.
